# Prediction of Staphylococcus aureus Antimicrobial Resistance by Whole-Genome Sequencing

**DOI:** 10.1128/JCM.03117-13

**Published:** 2014-04

**Authors:** N. C. Gordon, J. R. Price, K. Cole, R. Everitt, M. Morgan, J. Finney, A. M. Kearns, B. Pichon, B. Young, D. J. Wilson, M. J. Llewelyn, J. Paul, T. E. A. Peto, D. W. Crook, A. S. Walker, T. Golubchik

**Affiliations:** a NIHR Oxford Biomedical Research Centre, John Radcliffe Hospital, Oxford, United Kingdom; b Department of Infectious Diseases and Microbiology, Royal Sussex County Hospital, Brighton, United Kingdom; c Department of Mathematics and Statistics, University of Reading, Reading, United Kingdom; d Department of Microbiology, John Radcliffe Hospital, Oxford, United Kingdom; e Antimicrobial Resistance and Healthcare Associated Infections Reference Unit, Public Health England, Colindale, United Kingdom; f Public Health England, Royal Sussex County Hospital, Brighton, United Kingdom

## Abstract

Whole-genome sequencing (WGS) could potentially provide a single platform for extracting all the information required to predict an organism's phenotype. However, its ability to provide accurate predictions has not yet been demonstrated in large independent studies of specific organisms. In this study, we aimed to develop a genotypic prediction method for antimicrobial susceptibilities. The whole genomes of 501 unrelated Staphylococcus aureus isolates were sequenced, and the assembled genomes were interrogated using BLASTn for a panel of known resistance determinants (chromosomal mutations and genes carried on plasmids). Results were compared with phenotypic susceptibility testing for 12 commonly used antimicrobial agents (penicillin, methicillin, erythromycin, clindamycin, tetracycline, ciprofloxacin, vancomycin, trimethoprim, gentamicin, fusidic acid, rifampin, and mupirocin) performed by the routine clinical laboratory. We investigated discrepancies by repeat susceptibility testing and manual inspection of the sequences and used this information to optimize the resistance determinant panel and BLASTn algorithm. We then tested performance of the optimized tool in an independent validation set of 491 unrelated isolates, with phenotypic results obtained in duplicate by automated broth dilution (BD Phoenix) and disc diffusion. In the validation set, the overall sensitivity and specificity of the genomic prediction method were 0.97 (95% confidence interval [95% CI], 0.95 to 0.98) and 0.99 (95% CI, 0.99 to 1), respectively, compared to standard susceptibility testing methods. The very major error rate was 0.5%, and the major error rate was 0.7%. WGS was as sensitive and specific as routine antimicrobial susceptibility testing methods. WGS is a promising alternative to culture methods for resistance prediction in S. aureus and ultimately other major bacterial pathogens.

## INTRODUCTION

Whole-genome sequencing (WGS) is a rapidly advancing technology, and increasingly affordable benchtop sequencers could be in use in the routine clinical laboratory within the next decade (1). It may soon be practical to sequence specimens directly in a matter of hours, resulting in enormous diagnostic improvements and creating new challenges for the routine laboratory (2). One key application is likely to be antimicrobial resistance prediction from the genome sequence (“resistance genotype”), analogous to *in silico* multiplex PCR for a large number of known resistance genes. It is theoretically possible to recover the entire complement of genes encoding resistance from the genomic sequence of an isolate in a single step. Recently, Zankari et al. (3) and Stoesser et al. (4) reported on resistance prediction using a simple BLAST method. Despite the limited size and lack of independent validation in these studies, they provide intriguing hints that much phenotypic resistance may be simply explained using genotypic prediction from WGS.

To be used confidently in clinical practice, reliable genotypic prediction of antimicrobial resistance phenotype has to be demonstrated to the same standards as any new phenotypic method, using large diverse sets of unrelated isolates. Comprehensively validated genotypic prediction of antimicrobial resistance, ready for implementation in clinical practice, will require multiple large studies. However, early investigation of the performance of known genetic determinants for resistance prediction could establish the feasibility of this approach.

In this study, we describe a three-step approach for developing a resistance gene prediction method using Staphylococcus aureus, with (i) initial development using easily available bioinformatics algorithms and a “derivation set” of 501 isolates, (ii) testing and algorithm refinement, and (iii) validation of the method in a further unrelated set of 491 isolates.

## MATERIALS AND METHODS

### Creation of a catalogue of antimicrobial resistance genes.

A panel of antimicrobial agents (Tables 1 and 2) was identified for investigation based on those used routinely for management of S. aureus infections in the Oxford University Hospitals (OUH) National Health Service (NHS) Trust. To identify the genetic determinants encoding resistance to these antimicrobial agents, a literature search was conducted in PubMed using the medical subject heading (MESH) terms “Staphylococcus aureus” and “Drug resistance, microbial” and individual antimicrobial drug names to create a catalogue of antimicrobial resistance genes and variants, using published sequences deposited in GenBank (http://www.ncbi.nlm.nih.gov/GenBank/).

**TABLE 1:** Panel of genes associated with mobile genetic elements used for BLAST query^*a*^

Antimicrobial agent(s)	Gene^*b*^	Product^*c*^	Reference gene accession no. (nucleotide positions)
Penicillin	*blaZ*	Class A beta-lactamase	BX571856.1 (1913827–1914672)
Methicillin	*mecA*	Low-affinity PBP2	BX571856.1 (44919–46925)
Erythromycin	msrA*	Erythromycin resistance protein	CP003194 (54168–55634)
Erythromycin and clindamycin	*ermA*	rRNA adenine *N*-6-methyltransferase	BA000018.3 (56002–56733)
	*ermB*	rRNA adenine *N*-6-methyltransferase	AB699882.1 (4971–5708)
	*ermC*	rRNA adenine *N*-6-methyltransferase	HE579068 (7858–8592)
	*ermT*	23S rRNA methylase	HF583292 (11344–12078)
Tetracycline	*tetK*	MFS tetracycline effux pump	FN433596 (69118–70497)
	*tetL*	MFS tetracycline efflux pump	HF583292 (7713–9089)
	*tetM*	Ribosomal protection protein	CP002643 (427033–428952)
Vancomycin	*vanA*	Low-affinity peptidoglycan precursor	AE017171.1
Fusidic acid	*fusB*	Fusidic acid detoxification	CP003193.1 (1336–1977)
	far*	Ribosome protection protein	AY373761.1 (19072–19713)
Trimethoprim	*dfrA*	Insensitive dihydrofolate reductase	CP002120 (2093303–2093788)
	*dfrG*	Insensitive dihydrofolate reductase	FN433596 (502263–502760)
Gentamicin	*aacA-aphD*	6′-aminoglycoside *N*-acetyltransferase/2″-aminoglycoside phosphotransferase	FN433596.1 (2209531–2210970)
Mupirocin (high-level resistance)	*mupA*	Isoleucyl-tRNA synthetase	HE579068 (2157–5231)
	*mupB*	Isoleucyl-tRNA synthetase	JQ231224

a Presence of the gene correlates with phenotypic resistance. Reference sequences were obtained from published sequences from human clinical isolates.

b An asterisk indicates that the gene was added in v2.0.

c MFS, major facilitator superfamily.

**TABLE 2:** Panel of housekeeping genes with amino acid variants known to be associated with antimicrobial resistance^*a*^

Antimicrobial agent	Gene	Amino acid substitutions^*b*^	Reference gene accession no. (nucleotide positions)
Ciprofloxacin	*gyrA*	S84L, E88K, G106D, S85P, E88G, E88L	BX571857.1 (7005–9668)
	*grlA*	S80F, S80Y, E84K, E84G, E84V, D432G, Y83N, A116E, I45M, A48T, D79V, V41G, S108N	BX571857.1 (1386869–1389271)
	*grlB*	R470D*, E422D*, P451S*, P585S*, D443E*, R444S*	BX571857.1 (1384872–1386869)
Fusidic acid	*fusA*	A160V*, A376V, A655E, A655P*, A655V*, A67T*, A70V*, A71V*, B434N, C473S*, D189G*, D189V*, D373N*, D463G*, E233Q*, E444K, E444V*, E449K*, F441Y, F652S*, G451V, G452C, G452S, G556S, G617D, G664S, H438N, H457Q, H457Y, L430S*, L456F, L461K, L461S, M161I*, M453I, M651I, P114H, P404L, P404Q, P406L, P478S, Q115L, R464C, R464H, R464S, R659C, R659H, R659L, R659S, R76C*, S416F*, T385N, T387I*, T436I, T656K, V607I, V90A, V90I, Y654N*	BX571857.1 (577685–579766)
Rifampin	*rpoB*	A473T*, A477D, A477T*, A477V, D471G*, D471Y, D550G, H481D, H481N, H481Y, I527F, I527L*, I527 M*, ins 475H, ins G475*, L466S*, M470T*, N474K*, Q456K, Q468K, Q468L, Q468R, Q565R*, R484H, S463P, S464P, S486L, S529L*	BX571857 (568813–572364)
Trimethoprim	*dfrB*	F99Y, F99S, F99I, H31N, L41F, H150R, L21V*, N60I*	BX571857.1 (1464014–1464493)

a All housekeeping gene sequences were obtained from the genome of reference strain MSSA 476 (7).

b An asterisk indicates that the amino acid substitution was reported in association with other variants. For the expected effect of each variant/combination on MIC, please see the supplemental data. ins, insertion.

### Whole-genome sequencing, assembly, and resistance gene detection.

Ethical approval for sequencing S. aureus isolates from routine clinical samples and linkage to patient data without individual patient consent in Oxford and Brighton in the United Kingdom was obtained from Berkshire Ethics Committee (10/H0505/83) and the United Kingdom National Information Governance Board [8-05(e)/2010]. For all isolates, DNA was extracted and sequenced using the Illumina HiSeq 2000 platform (San Diego, CA, USA) as previously described (5). To assess sequencing quality, reads were mapped to reference strain MRSA 252 (GenBank accession no. BX571856.1) using Stampy v1.0.18 (6). MRSA 252 was chosen as it contains staphylococcal cassette chromosome *mec* (SCC*mec*), has been capillary sequenced (7), and belongs to a common United Kingdom methicillin-resistant S. aureus (MRSA) clone (EMRSA 16). To obtain whole genomes for BLAST, reads were then *de novo* assembled using Velvet v1.0.18 (8). Samples were excluded if they failed quality checks either on mapping (<70% coverage of reference genome after filtering) or assembly (<50% of the genome in contigs > 1 kb).

### Initial method development using the derivation set.

The *de novo*-assembled genomes were interrogated with BLAST+ (v 2.2.28+) blastn and tblastn (9) to identify nucleotide sequences matching genes from the panel and their matching protein sequences, respectively. The parameters for the two programs were as follows: for blastn, word size of 17, gap opening penalty of 5, and gap extension penalty of 2; for tblastn, word size of 3, gap opening penalty of 11, and gap extension penalty of 1. The E-value cutoff was set at 0.001. Relative coverage was defined as the product of the proportion of reference allele matched and the sequence identity of the match. For the initial algorithm (v1.0), a relative coverage threshold of >80% was chosen to define gene presence with a high degree of similarity to the reference, based on pilot data (for example, 95% relative coverage may be 95% of the gene length with 100% identity or 100% of the gene length with 95% identity). For housekeeping genes, where resistance is conferred by one or more point mutations, differences between the tblastn result and the query protein sequence were compared to the sequence of the wild-type protein and compared against the catalogue of known antimicrobial resistance-encoding mutations compiled above. Changes in protein sequence (at the same or different codons) which were not previously reported as conferring resistance were counted as susceptible.

To determine the diversity of the isolates tested, *in silico* prediction of multilocus sequence type (MLST) was also performed using BLAST+. The S. aureus MLST alleles were extracted from assemblies based on sequence similarity to allele 1 for each locus, and the online MLST database (http://saureus.mlst.net/) was used to predict the ST.

The initial development of the algorithm was not done in a blind manner, using 501 clinical S. aureus isolates which had been sequenced and phenotyped previously and whose WGS and resistance data were available (“derivation set”). To ensure a representative range of sequence types, isolates were identified from bacteremia and carriage collections held at the Oxford Radcliffe Hospitals NHS Trust and Brighton and Sussex University Hospitals NHS Trust, spanning a period of 13 years (Table 3) (10). The collection included 159 MRSA isolates (32%). All isolates had been tested at each site by the routine clinical laboratories for resistance to a standard first-line panel of antimicrobial agents (penicillin, methicillin, erythromycin, vancomycin, ciprofloxacin, tetracycline, gentamicin, fusidic acid, and rifampin at both sites; mupirocin and clindamycin for Brighton isolates only; trimethoprim for Oxford isolates only). In the Brighton clinical laboratory, susceptibility testing was performed using the Vitek automated system (bioMérieux, Basingstoke, United Kingdom), and in the Oxford clinical laboratory, isolates were phenotyped by disc diffusion (11). The susceptibility testing results were retrieved electronically from laboratory databases. Methicillin resistance was tested using cefoxitin (Brighton) or oxacillin (Oxford).

**TABLE 3:** Source of isolates and method of susceptibility testing

Collection	No. of isolates	Source^*a*^	Specimen type	Dates	Susceptibility testing methods	Sequencing
Derivation set collections (*n* = 501)	88	Brighton (all wards)	Blood	1999–2007	Automated broth dilution (Vitek), clinical laboratory	Previously sequenced
	90	Brighton carriage (ITU)	Nasal swab	2010–2011	Disc diffusion, clinical laboratory	Previously sequenced
	323	Oxford (all wards)	Blood	2008–2011	Disc diffusion, clinical laboratory	Previously sequenced
Validation set collections (*n* = 491)	102	Oxford carriage (ITU)	Nasal swab	2009	Disc diffusion and automated broth dilution (BD Phoenix)	Previously sequenced
	100	Oxford carriage (community)	Nasal swab	2009	Disc diffusion and automated broth dilution (BD Phoenix)	Previously sequenced
	165	Brighton (all wards)	Blood	2011–2012	Disc diffusion and automated broth dilution (BD Phoenix)	Sequenced *de novo*, susceptibility testing performed from same subculture as sequencing
	124	Oxford (all wards)	Blood	2011–2012	Disc diffusion and automated broth dilution (BD Phoenix)	Sequenced *de novo*, susceptibility testing performed from same subculture as sequencing

a Brighton, Brighton and Sussex University Hospitals NHS Trust; Oxford, Oxford Radcliffe Hospitals NHS Trust; ITU, intensive therapy unit.

### Comparison of phenotype and predicted genotypic resistance.

The predicted susceptibilities based on mobile and chromosomal genetic elements, using the whole-genome sequences of the isolates in the derivation set, were compared with the routine laboratory susceptibility testing results. Where there was a mismatch between genotypic prediction and recorded phenotype, isolates were retrieved from storage at −80°C and had repeat susceptibility tested by gradient diffusion using EUCAST breakpoints (http://www.eucast.org/clinical_breakpoints/) to resolve the phenotype (“discordant repeat”). A very major error (VME) was defined as a susceptible genotype with a resistant phenotype, and a major error (ME) was defined as a resistant genotype with a susceptible phenotype.

### Revision of bioinformatics algorithm.

Using the results obtained above, we further examined genotype-phenotype mismatches to identify whether algorithm improvements could be made. We extended the gene panel by manually searching references from the original search and added two additional genes to the panel (*msrA* and *far*) and added to the list of variants for *fusA*. We noted a high VME rate for penicillin and fusidic acid which was reduced by adjusting the algorithm quality filters to accept short or low-coverage contigs for *blaZ*, *fusB*, and *far* (see Results for details). We estimated sensitivity and specificity with different thresholds for the relative coverage required for these genes to be considered present in the derivation set. For the revised algorithm (v2.0), the best compromise between overall sensitivity and specificity was obtained by defining resistance as >30% relative coverage for *blaZ*, *fusB*, and *far* and as >80% relative coverage for the remaining mobile genes.

### Blind validation of revised prediction method v2.0.

Because repeat susceptibility testing and revision of the genotype prediction algorithm were done with *a priori* knowledge of the phenotype, to validate the method, we applied v2.0 to a further 491 isolates (the “validation set” [Table 3]), with no previous information regarding the expected phenotype available before genotypic interrogation (i.e., blind to phenotype).

A total of 202 isolates were sourced from carriage collections (12) which had previously been sequenced but not phenotyped. These isolates were retrieved from storage at −80°C for resistance testing. A further 289 isolates were obtained from archived bloodstream collections at the Oxford and Brighton sites. For these, single colonies were plated on Columbia blood agar and grown at 37°C for 18 to 24 h. All the bacteria on the plate were harvested and suspended in 1.5 ml physiological saline. A portion (0.1 ml) was removed and replated onto Columbia blood agar and incubated overnight at 37°C for resistance phenotyping, and the remaining suspension was used to prepare DNA for WGS.

To control for differences in phenotypic testing methods, all 491 isolates had antimicrobial susceptibility testing performed by M. Morgan using the Phoenix automated microbiology system (BD Biosciences, Sparks, MD, USA) for a standard panel of antimicrobial agents (penicillin, methicillin, ciprofloxacin, erythromycin, fusidic acid, gentamicin, mupirocin, rifampin, tetracycline, and vancomycin). Isolates were also tested by disc diffusion (13) by K. Cole and J. R. Price (Brighton isolates, panel as described above) and N. C. Gordon (Oxford isolates, panel as described above plus trimethoprim). Concordant results for the two methods were used as the final phenotype. Isolates that were resistant to erythromycin were further tested for clindamycin resistance by disc diffusion and the clindamycin D test (14). Where there was discordance between disc diffusion and BD Phoenix, repeat testing was performed by the gradient diffusion method (Etest) using EUCAST breakpoints, and this value was used as the phenotype.

Whole-genome sequences were interrogated by BLAST+ using the revised parameters as described above, performed in a blind manner (the phenotype not known) by T. Golubchik. Finally, the phenotypic and genotypic profiles were compared by a separate investigator (T. E. A. Peto). The sensitivity, specificity, and error rates (calculated as percentages of the total number of resistance tests) were calculated against the concordant phenotype.

As in the derivation set, where there was a mismatch between the genotype and phenotype, isolates had repeat susceptibility tested by gradient diffusion (“discordant repeat”). Possible explanations for the remaining discordant results were explored by testing for penicillinase production with nitrocefin discs (15) and manually inspecting sequences for the presumed resistance-encoding genes or mutations to give an amended algorithm for future testing (v2.1).

### Study accession number.

The sequences reported in this paper have been deposited in the European Nucleotide Archive Sequence Read Archive under study accession number ERP004655.

## RESULTS

### Derivation set.

**FIG 1:**
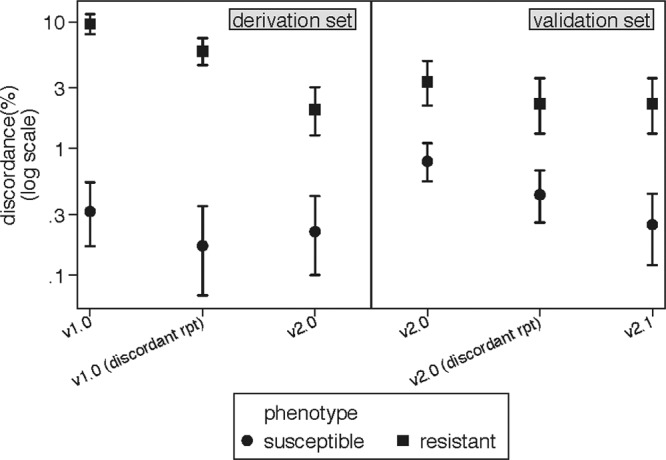
Comparison of percentages of errors for the derivation and validation sets, illustrating the change in error rate with repeat phenotyping and with optimized algorithm versions. Error rates for resistant and susceptible isolates are shown for each step of algorithm development: in the derivation set, the error rate was decreased by repeat susceptibility testing (discordant rpt). The VME rate was reduced by adjusting the algorthm parameters (v2.0), although this resulted in a slight increase in the ME rate. In the validation set, error rates were relatively low to start with and were improved further by repeat testing of the discordants (v2.0 discordant rpt) and by incorporating the novel *blaZ* mutations (v2.1).

WGS and routine laboratory antimicrobial susceptibility testing results were available for 506 isolates, with 46 different sequence types found by *in silico* MLST. Using the initial v1.0 algorithm, 439 isolates (87%) had complete concordance between the genotype and phenotype for all antimicrobial agents tested. The remaining 67 isolates had a total of 123 discrepancies. For five of these isolates, the frozen bacterial stocks were found to be contaminated with other organisms (one isolate with Proteus sp. and four isolates with coagulase-negative staphylococci), and further confirmatory testing could not be performed. These isolates were excluded from further analysis, leaving 501 isolates in the derivation set. Repeating the susceptibility testing for the isolates with phenotype/genotype mismatches reduced the error rate, with a total of 71 discrepancies in the 49 isolates remaining (Fig. 1).

We noted a high VME rate for penicillin, with 9 (1.8%) isolates apparently resistant to penicillin (MIC > 0.12 mg/liter) in the absence of the *blaZ* gene based on >80% relative coverage in WGS data. However, all 9 isolates were positive for *blaZ* by PCR. On manual inspection of the sequences, we found that the *blaZ* gene was in fact present on small sequence contigs (<300 bp) or contigs with low coverage (depth of cover < 5) which did not meet the algorithm quality standard and had therefore been excluded. Including these short or low-coverage contigs in the algorithm (v2.0) improved predictions (with relative coverage as low as 30% associated with phenotypic resistance). This phenomenon was also noted for fusidic acid, where the inclusion of low-quality contigs for the *fusB* and *far* genes improved prediction of phenotype. This was not the case for the remaining genes associated with mobile elements in the panel where lowering the relative coverage threshold substantially increased false-positive results.

Using this revised genotypic prediction method (v2.0), we found 31 persistent discrepancies in 28 isolates across all antimicrobials in the derivation set (Fig. 1 and Table 4). There were 22 very major errors (0.4% VME rate) and 9 major errors (0.2% ME rate). The overall sensitivity and specificity were 0.98 (95% confidence interval [95% CI], 0.97 to 0.99) and 1.00 (95% CI, 0.99 to 1.00), respectively.

**TABLE 4:** Derivation set results^*a*^

Antimicrobial agent	No. of isolates resistant by phenotype		No. of isolates susceptible by phenotype		Total no. of isolates	Very major error rate (%)	Major error rate (%)	Sensitivity (95% CI)	Specificity (95% CI)
	Susceptible by genotype	Resistant by genotype	Susceptible by genotype	Resistant by genotype					
Penicillin	4	438	59	0	501	0.8	0	0.99 (0.98–1.00)	1.00 (0.92–1.00)
Methicillin	1	158	341	1	501	0.2	0.2	0.99 (0.96–1.00)	1.00 (0.98–1.00)
Ciprofloxacin	7	165	326	3	501	1.4	0.6	0.96 (0.91–0.98)	0.99 (0.97–1.00)
Erythromycin	1	133	366	1	501	0.2	0.2	0.99 (0.95–1.00)	1.00 (0.98–1.00)
Clindamycin	0	88	89	0	177^*c*^	0	0	1.00 (0.95–1.00)	1.00 (0.95–1.00)
Tetracycline	0	28	473	0	501	0	0	1.00 (0.85–1.00)	1.00 (0.99–1.00)
Vancomycin	0	0	501	0	501	0	0	N/A^*d*^	1.00 (0.99–1.00)
Fusidic acid	3^*b*^	38	458	2	501	0.6	0.4	0.93 (0.79–0.98)	1.00 (0.98–1.00)
Trimethoprim	5	10	308	0	323	1.5	0	0.67 (0.39–0.87)	1.00 (0.98–1.00)
Gentamicin	0	7	494	0	501	0	0	1.00 (0.60–1.00)	1.00 (0.99–1.00)
Mupirocin	0	2	174	2	178	0	1.1	1.00 (0.20–1.00)	0.99 (0.96–1.00)
Rifampin	1	2	498	0	501	0.2	0	0.67 (0.13–0.98)	1.00 (0.99–1.00)
Overall	22	1,069	4,087	9	5,187	0.2	0.4	0.98 (0.97–0.99)	1.00 (0.99–1.00)

a Comparison of results for individual antimicrobial agents for 501 carriage/bacteremia isolates by phenotype (Vitek or disc diffusion) and predicted susceptibility using v2.0 genotypic prediction method. The result (resistant or susceptible) by phenotype refers to Vitek or disc diffusion results, and the result by genotype refers to the predicted susceptibility using the v2.0 genotypic prediction method.

b Two isolates had two nonsynonymous mutations in *fusA* not previously described in the literature (T326I plus E468V and T326I plus V90I) which may be responsible for the observed phenotypes.

c One isolate failed to grow for clindamycin testing.

d N/A, not applicable.

### Blind validation set and genotypic prediction method v2.0.

For the 491 isolates in the validation set, 61 different sequence types were identified by *in silico* MLST. The most frequent sequence types were ST22 (13%) and ST30 (12%). Fifty-seven isolates were MRSA (12%). There were 60 errors in 48 isolates out of 5,193 antimicrobial susceptibilities tested (Fig. 1 and Table 5). There were 25 VMEs: 6 for ciprofloxacin, 4 for erythromycin, 2 for clindamycin, 4 for fusidic acid, 3 for penicillin, 2 for methicillin, 2 for gentamicin, and 2 for trimethoprim. Of the 35 major errors, 25 were for penicillin. The very major error rate was 0.5%, and the major error rate was 0.7%. The overall sensitivity was 0.97 (95% CI, 0.95 to 0.98), and the overall specificity was 0.99 (95% CI, 0.98 to 1). Further details for each of the isolates with discrepant results are given in Tables 6 (penicillin) and 7 (other antimicrobial agents).

**TABLE 5:** Validation set results^*a*^

Antimicrobial agent	No. of isolates resistant by phenotype		No. of isolates susceptible by phenotype		Total no. of isolates	Very major error rate (%) (95% CI)	Major error rate (%) (95% CI)	Sensitivity (95% CI)	Specificity (95% CI)
	Susceptible by genotype^*b*^	Resistant by genotype	Susceptible by genotype	Resistant by genotype^*b*^					
Penicillin	3 (2)	379	84	25 (9)	491	0.6 (0.1–1.8)	5.1 (3.3–7.4)	0.99 (0.98–1.00)	0.77 (0.68–0.84)
Methicillin	2 (1)	55	432	2 (1)	491	0.4 (0.05–1.5)	0.4 (0.05–1.5)	0.96 (0.87–0.99)	1.00 (0.98–1.00)
Ciprofloxacin	6 (4)	64	420	1 (0)	491	1.2 (0.4–2.6)	0.2 (0.05–1.1)	0.91 (0.82–0.96)	1.00 (0.98–1.00)
Erythromycin	4 (2)	79	405	3 (3)	491	0.8 (0.2–2)	0.6 (0.1–1.8)	0.95 (0.87–0.98)	0.99 (0.98–1.00)
Clindamycin	2 (2)	77	2	0	81	2.5 (0.3–8.6)	0.0 (0–4.4)	0.97 (0.90–1.00)	1 (0.20–1.00)
Tetracycline	0	18	471	2 (2)	491	0.0 (0–0.7)	0.4 (0.05–1.5)	1.00 (0.78–1.00)	1.00 (0.98–1.00)
Vancomycin	0	0	491	0	491	0.0 (0–0.7)	0.0 (0–0.7)	N/A^*c*^	1.00 (0.99–1.00)
Fusidic acid	4 (4)	39	448	0	491	0.8 (0.2–2)	0.0 (0–0.7)	0.91 (0.77–0.97)	1.00 (0.99–1.00)
Trimethoprim	2 (2)	1	197	2 (1)	202	1.0 (0.1–3.5)	1.0 (0.1–3.5)	0.33 (0.02–0.87)	0.99 (0.96–1.00)
Gentamicin	2 (2)	2	487	0	491	0.4(0.05–1.5)	0.0 (0–0.7)	0.50 (0.09–0.91)	1.00 (0.99–1.00)
Mupirocin	0	2	489	0	491	0.0(0–0.7)	0.0 (0–0.7)	1.00 (0.20–1.00)	1.00 (0.99–1.00)
Rifampin	0	5	486	0	491	0.0 (0–0.7)	0.0 (0–0.7)	1.00 (0.46–1.00)	1.00 (0.99–1.00)
Overall	25 (19)	644	4,410	35 (16)	5,112	0.5 (0.3–0.7)	0.7 (0.5–0.9)	0.97 (0.95–0.98)	0.99 (0.99–1.00)

a Comparison of susceptibility results for 491 bacteremia and carriage isolates by phenotype (Phoenix/disc diffusion consensus result) and genotype prediction tool v2.0. The result (resistant or susceptible) by phenotype refers to Phoenix or disc diffusion consensus results, and the result by genotype refers to the predicted susceptibility using the v2.0 genotypic prediction method.

b Figures in parentheses are numbers of isolates with discrepant phenotype confirmed on repeat testing.

c N/A, not applicable.

**TABLE 6:** Results for MICs, nitrocefin disc testing, and *blaZ* variants for isolates with discrepant results for penicillin in the validation set by the v2.0 genotype prediction method

Isolate^*a*^	*In silico* MLST	Initial consensus phenotype^*b*^	MIC (mg/liter)	Nitrocefin disc test result		*blaZ* variant^*c*^
				10 min	120 min	
C00001124	ST45	S	0.12	−	−	InsA436
C00001241	ST30	S	0.12	−	−	InsA256
C00001144	ST30	S	0.12	−	−	A99−
C00001203	ST30/36	S	0.06	−	−	A99−
C00013228	ST30	S	0.06	−	−	A99−
C00013375	ST7	S	0.12	−	−	A99−
C00001080*	ST22	S	0.12	+	+	Similar to wild type
C00001092*	ST2417	S	0.12	+	+	Similar to wild type
C00001112	ST2438	S	0.25	+	+	Similar to wild type
C00001148	ST1	S	0.12	+	+	Similar to wild type
C00001158	ST582	S	0.12	+	+	Similar to wild type
C00001142	ST582	S	0.12	+	+	Similar to wild type
C00001192	ST2417	S	0.12	+	+	Similar to wild type
C00001199	ST30	S	0.12	+	+	Similar to wild type
C00001205	ST30	S	0.06	+	+	Similar to wild type
C00001217	ST30	S	0.12	+	+	Similar to wild type
C00001231	ST22	S	0.12	+	+	Similar to wild type
C00001266	ST188	S	0.12	+	+	Similar to wild type
C00012754	ST20	S	0.25	+	+	Similar to wild type
C00001277	ST2445	S	0.12	−	+	Similar to wild type
C00013249	ST45	S	0.06	−	+	Similar to wild type
C00001093	ST15	S	0.12	−	+	Similar to wild type
C00001104	ST8	S	0.023	−	−	Similar to wild type
C00001111	ST5	S	0.12	−	−	Similar to wild type
C00001182	ST5	S	0.12	−	−	Similar to wild type
C00001147*	ST22	R	0.5	−	−	Absent
C00012780	ST97	R	0.25	−	−	Absent
C00013232	ST7	R	0.06	−	−	Absent

a Isolates with discrepancies for one or more other antimicrobial agents are indicated by an asterisk.

b S, sensitive; R, resistant.

c InsA436, insertion of A at position 436; A99−, deletion of A at position 99.

**TABLE 7:** Details of antimicrobial discrepancies for validation set and genotype prediction tool v2.0

Type of error and antimicrobial agent(s)	Isolate^*a*^	Initial consensus phenotype^*b*^	MIC (mg/liter) and/or phenotype on repeat testing^*b*^	Genotyping details
Very major errors				
Methicillin	C00001115*#	R	0.5 (S)	No *mecA* or *mecC* detected
	C00001162*	R	16	No *mecA* or *mecC* detected
Ciprofloxacin	C00013185	R	1.5	No significant *gyrA* or *grlA* mutation
	C00001162*	R	2	No significant *gyrA* or *grlA* mutation
	C00001092*	R	2	No significant *gyrA* or *grlA* mutation
	C00001115*	R	2	No significant *gyrA* or *grlA* mutation
	C00001105#	R	<0.125 (S)	No significant *gyrA* or *grlA* mutation
	C00001109#	R	<0.125 (S)	No significant *gyrA* or *grlA* mutation
Erythromycin	C00001147#	R	0.75 (S)	No *erm* or *msrA* gene detected
	C00013384#	R	0.75 (S)	No *erm* or *msrA* gene detected
Erythromycin/clindamycin	C00001224	R/R	2/ND	No *erm* or *msrA* gene detected
	C00001162*	R/R	3/ND	No *erm* or *msrA* gene detected
Fusidic acid	C00013212	R	R (disc only)	V90I mutation in *fusA*
	C00013194	R	R (disc only)	V90I mutation in *fusA*
	C00001259	R	R (disc only)	Wild-type *fusA*, no *fusB* or *far* detected
	C00001276	R	R (disc only)	T656S substitution in *fusA*
Trimethoprim	C00001092*#	R	S (disc only)	Wild-type *dfrB*, no *dfrA* or *dfrG* detected
	C00001235#	R	S (disc only)	Wild-type *dfrB*, no *dfrA* or *dfrG* detected
Gentamicin	C00001115*	R	24	No *aacA-aphD*, *aadD* or *aadE*, or *aphA3* detected
	C00013331	R	3	No *aacA-aphD*, *aadD* or *aadE*, or *aphA3* detected
Major errors				
Methicillin	C00001222#	S	16 (R)	*mecA* gene detected, 100% relative coverage
	C00001080*	S	0.3	*mecA* gene detected, 100% relative coverage
Ciprofloxacin	C00001080*#	S	2 (R)	S80F mutation in *grlA*, S84l mutation in *gyrA*
Erythromycin	C00001249	S	0.25	*ermA* gene detected, 100% relative coverage
	C00001189	S	0.25	*ermC* gene detected, 100% relative coverage
	C00001080*	S	0.5	*ermC* gene detected, 100% relative coverage
Tetracycline	C00012796	S	0.19	*tetK* gene detected, 100% relative coverage
	C00001247	S	0.75	*tetM* gene detected, 95% relative coverage
Trimethoprim	C00001240	S	S (disc only)	H31N mutation in *dfrB*, usually confers resistance
	C00001123#	S	R (disc only)	*dfrG* gene detected, 100% relative coverage

a Isolates with discrepancies for two or more antimicrobial agents are indicated by an asterisk after the isolate name. Isolates for which the phenotype matched the genotype on repeat testing are indicated by a hash symbol (#) after the isolate name.

b R, resistant; S, sensitive. Erythromycin and clindamycin were tested; the first phenotype or MIC is for erythromycin, and the second is for clindaymycin. ND, not done.

### (i) Penicillin.

A total of 382 isolates were penicillin resistant (78%). Of the 3 very major errors (Table 5), one isolate was susceptible on repeat testing (MIC of 0.06 mg/liter), while the other 2 were confirmed as resistant by Etest (MICs of 0.5 mg/liter and 0.25 mg/liter).

Although by using the prototype genotypic prediction method, we found a high rate of very major errors for penicillin, using the lower relative coverage (>30%) threshold to define resistance for v2.0 resulted in a high rate of major errors (5%), illustrating the inherent trade-off between sensitivity and specificity for any algorithm.

Of the 25 isolates with major errors (*blaZ* positive but susceptible by initial phenotyping), 2 were penicillin resistant when retested by Etest (MICs of 0.25 mg/liter) and were also positive for penicillinase production. Eleven isolates were positive for penicillinase production at 10 min, and a further 3 isolates were weakly positive (i.e., positive after 2 h). Nine remaining isolates with *blaZ* had no penicillinase activity on nitrocefin disc testing and had susceptible MICs by gradient diffusion (≤0.12 mg/liter). Inspection of the *blaZ* genes found single-base-pair insertions in 2 cases (positions 256 and 436, respectively, relative to the reference sequence), causing a frameshift in the translated protein which may explain the lack of enzymatic activity. Similarly, 4 further isolates had identical deletions at position 99, again resulting in a frameshift and premature termination and correlating with a complete absence of pencillinase activity. We did not find any of these mutations in the *blaZ*-positive, fully phenotypically resistant isolates. Revising the algorithm for *blaZ* alone to define presence (>30% coverage) of *blaZ* with these insertions/deletions as susceptible (v2.1), only 3 isolates had wild-type *blaZ* with no penicillinase production detected by any of the phenotypic test methods, giving a major error rate of 0.6% which is in keeping with the observed error rates for the other antimicrobial agents in this study.

### (ii) Methicillin.

Fifty-seven isolates (12%) were MRSA by initial phenotype. We found 2 very major errors (0.4%) and 2 major errors (0.4%) for methicillin. Of the very major errors, one isolate was susceptible on repeat testing (MIC of 0.5 mg/liter), and similarly, one of the isolates with a major error was resistant on repeat testing (MIC of 16 mg/liter), reflecting either sample heterogeneity or more probably laboratory error. Both methicillin resistance in the absence of *mecA* resulting from overexpression of penicillinase (16) and methicillin susceptibility despite the presence of *mecA* have been previously described (5, 17), potential explanations for the 2 remaining errors. None of the isolates contained *mecC* (18).

### (iii) Ciprofloxacin.

Seventy isolates were phenotypically resistant (14%). In both derivation and validation sets, the highest VME rate was seen for ciprofloxacin. For the validation set, this was 6/491 (1.2%), with a major error rate of 0.2% (1/491). Two of the 6 isolates with very major errors were susceptible on repeat testing. For the four other isolates with discrepant results, we examined the *grlB*, *gyrB*, and *norA* genes but found no significant mutations (19, 20). The single isolate with a major error was found to be resistant on repeat testing (MIC of 2 mg/liter). Of the 64 isolates predicted to be resistant by genotype and resistant by phenotype, 60 had the amino acid substitutions S80F in *grlA* and S84L *in gyrA*. Four isolates had S84L in *grlA* only.

### (iv) Erythromycin and clindamycin.

A total of 83 (17%) isolates were resistant to erythromycin by phenotype. For these 83 isolates, we observed 4 very major errors (0.8%). Two of these were susceptible on repeat testing (MICs of 0.75 mg/liter), but the other 2 were confirmed as resistant. There were 3 major errors (0.6%) which were all confirmed as susceptible (MICs, 0.25 to 5 mg/liter). Two had *ermC* detected by BLAST, and one had *ermA*. For the concordant resistant isolates, 37 had *ermA* alone, 37 had *ermC* alone, 1 had *ermT*, 2 had *msrA* alone, 1 had *ermC* and *msrA*, and 1 had *ermC* and *ermA*. None of the isolates had *ermB*. Out of 81 confirmed erythromycin-resistant isolates, 45 were resistant to clindamycin by disc diffusion and 36 were susceptible. Of these isolates, 34 had inducible resistance by D-test and contained either *ermA*, *ermC*, or *ermT* as described above. The two isolates that were resistant to erythromycin but susceptible to clindamycin contained *msrA* only. There were 2 very major errors (same isolates as for erythromycin VMEs). We did not observe any correlation between *erm* variant and inducibility of clindamycin resistance. We did not find *vga*(A)_LC_ in any of the isolates (21).

### (v) Tetracycline.

Eighteen isolates were phenotypically resistant (4%). Two major errors were seen for tetracycline (0.4%), and both isolates were confirmed susceptible by Etest. One isolate had *tetK* detected by BLAST (MIC of 0.19 mg/liter), and the other had *tetM* (MIC of 0.75 mg/liter).There were no very major errors. Of the 18 concordant resistant isolates, 16 had *tetK*, 1 had both *tetK* and *tetL*, and 1 had *tetM*.

### (vi) Vancomycin.

Vancomycin resistance was not identified in our isolates either phenotypically or genotypically. Consequently, although the specificity of the method was 1.00, its sensitivity is not estimable due to the rarity of *vanA*-mediated resistance. To investigate the possibility of intermediate resistance missed by phenotyping, *post hoc* we also screened the collection for recently published mutations in the *yycG* gene (22) found to be associated with intermediate MICs in laboratory mutants but did not identify these mutations in any isolate.

### (vii) Fusidic acid.

Forty-three isolates were phenotypically resistant (9%). Four very major errors were observed for fusidic acid. In 2 cases, manual examination of the sequences revealed single point mutations in the chromosomal *fusA* gene, predicted to result in the amino acid substitution of isoleucine for valine at position 90. This substitution has been reported in both susceptible and resistant isolates (23), and therefore, its role in resistance is unresolved. One resistant isolate had a substitution of serine for threonine at position 656 (predicted sensitive on genotyping based on a review of the literature). Since the substitution of lysine for threonine at the same position is associated with full phenotypic resistance (23), this may explain the discrepancy seen in this case.

### (viii) Trimethoprim.

Phenotypic testing for trimethoprim resistance was performed for only 202 isolates, as it is not routinely tested in Brighton, and it was not part of the BD Phoenix panel used. Results are therefore taken from Oxford isolates, tested by disc diffusion testing only. Three isolates were phenotypically resistant (2%). There were 2 very major errors (1%) and 2 major errors. Both isolates with very major errors were susceptible on repeat testing (performed by disc diffusion only), and 1 isolate with a major error was resistant on repeat testing.

### (ix) Gentamicin.

Four isolates were phenotypically resistant (1%). There were 2 very major errors, and resistance was confirmed by repeat testing (MICs of 24 mg/liter and 3 mg/liter). We did not find *aacA-aphD* in either isolate, and they were also negative for *aadD*, *aadE*, and *aphA3*. The low frequency of resistance in this collection means the evaluation is underpowered, which makes accurately estimating the overall sensitivity impossible.

### (x) Mupirocin and rifampin.

Two isolates (0.4%) and 5 (1%) isolates were resistant to mupirocin or rifampin, respectively. There were no errors observed; however, the relatively low resistance rates limit the power of the evaluation. Mupirocin resistance in both cases was attributable to *mupA*.

## DISCUSSION

We have developed and tested a method for genotypic prediction of antimicrobial susceptibility phenotype from whole-genome sequencing within a single species, using substantial derivation (*n* = 501) and validation (*n* = 491) sets. Our goal was to develop a method with direct relevance to clinical practice, and therefore, our prediction tool is based on genetic mechanisms that have been reported in clinical isolates, rather than including the very large number of potential resistance determinants in existing databases such as ResFinder (24) and CARD (Comprehensive Antibiotic Resistance Database) (25), many of which have not been phenotypically verified in clinical isolates. Excluding phenotypic errors and isolates with multiple discrepancies, the final results show highly promising concordance, with a very major discrepancy rate of 8/652 (1.2%) and a major discrepancy rate of 13/4,423 (0.3%), comparable with the error rates for current phenotypic methodologies (26, 27) and within the acceptable limits set by the U.S. Food and Drug Administration (28) for marketing approval of new susceptibility testing methods (<1.5% very major discrepancy rate, <3% major discrepancy rate).

In both the derivation and validation sets, a substantial number (40%) of the initial observed errors were resolved by repeat phenotypic susceptibility testing. This is shown in Fig. 1, which illustrates the phenotype/genotype discrepancies on initial testing, after repeat phenotyping, and after adjustment of the algorithm for both the derivation and validation sets. Even when testing according to published guidelines, occasional errors in reagent and medium storage, incubation conditions, and inoculum density may contribute to variation in observed phenotype, and similarly, inaccuracies in labeling, interpretation, and data entry are liable to occur in any system which is not fully automated. Some of these factors may also contribute to the genotyping results, and this is supported by the fact that several isolates were discordant for multiple antimicrobial agents, suggesting a labeling or storage error.

The most problematic antimicrobial agent was penicillin, with an unacceptably high very major error rate (1.8%) using v1.0 in the derivation set and an unacceptably high major error rate (5%) in the validation set using v2.0. This may be due to the variable location of *blaZ*, which may be on a plasmid or integrated into the chromosome (29). Isolates with chromosomally integrated *blaZ* are likely to have average coverage in the sequencing reads, while isolates with plasmid-carried copies may have very high (if multiple copies are carried) or very low (because of poor mapping to the reference) coverage in that region. As a result, these regions may be rejected as poor quality by the assembly software, because they fall outside the coverage levels of the rest of the genome. This problem may be overcome in future with longer reads or alternative methods for *de novo* assembly; however, our results highlight that relative coverage cutoff values may need to be set individually based on gene location. Conversely, in the validation set, we found that most of the major errors were due to a lack of concordance between penicillinase production and disc or broth dilution testing (30). We also identified three novel *blaZ* frameshift mutations that were associated with susceptibility despite the presence of the gene. Similar frameshift deletions have been reported in *blaZ*-positive, penicillin-susceptible isolates from cows with mastitis (31).

Relatively high very major error rates (1.2%) were also seen for ciprofloxacin. Staphylococcal resistance to quinolones is predominantly due to point mutations in the *grlA* and *gyrA* genes (19). Low-level resistance may result from mutations in *grlB* or *gyrB* or alterations in expression of the efflux pump NorA (20). However, in most cases, these are in combination with *grlA* or *gyrA* mutations, and consequently, the overall phenotypic effect is difficult to predict. All isolates in this study contained the *norA* gene, but no correlation was seen between quinolone resistance and the *norA* gene or its known regulators, or the *grlB* or *gyrB* gene. Further studies may be able to elucidate the contribution of these to overall quinolone resistance in S. aureus.

There remains a very small subset of isolates where the genetic basis for an antimicrobial phenotype of the organism was not clear (15/48, excluding presumed phenotyping errors or identified novel variants). If not due to human error, these may be due to sequencing or assembly error, for example a miscalled base at a critical position. However, for S. aureus, our group has sequenced multiple replicates of strain MRSA 252 for comparison with a capillary sequenced high-quality reference sequence of this genome (GenBank accession no. BX571856.1). The in-house estimated false-positive rate (detection of a spurious variant) for our bioinformatics pipeline was previously estimated to be 2.5 × 10^9^ per nucleotide, i.e., 0.0075 per genome (32). Consequently, although an incorrect susceptibility prediction may in theory occur because of a sequencing error, in practice we anticipate that the impact of this will be extremely small.

A more likely explanation for the discrepancies may be as yet unidentified alterations in regulatory regions or alternative resistance mechanisms. This highlights a major challenge for *in silico* resistotyping, since a query-based method cannot recognize novel variants that are not in the relevant database. Furthermore, gene expression is the result of complex interactions between transcription promoters, repressors, and other regulatory molecules, which may be remotely located from the gene itself. Phenotypic assays address these challenges by measuring the overall combined impact of all these mechanisms, although it is important to recognize that factors resulting in delayed transcription may cause isolates to be falsely identified as susceptible, as we found for penicillin.

Therefore, it is unlikely that WGS will be able to replace phenotypic methods entirely, and some form of phenotypic surveillance will need to be maintained, for example based on clusters of treatment failures. However, the need for routine phenotyping should diminish as examination of WGS and phenotyping data for isolates with apparently novel mutations or with nonfunctioning resistance genes elucidate the contribution of the underlying genetics. This new knowledge can then be added to resistance determinant databases and absorbed into WGS investigations, as demonstrated by the improved specificity resulting from the incorporation of the novel *blaZ* variants above into the algorithm. The huge potential of WGS data lies in its completeness: once sequenced, a genome can be accessed repeatedly to query for novel genes of interest as these arise (e.g., by our scan for recently published *yycG* gene mutations [22]).

The cost and turnaround time for WGS have fallen rapidly in recent years, with the current full economic cost of sequencing a single isolate estimated to be less than £40 ($65) (33) compared with approximately £5 ($8) per sample using the BD Phoenix. Current turnaround times are directly comparable, with next-generation sequencers able to deliver results in 27 h (5) and the likelihood that this will be reduced to a matter of hours in the near future. The potential for full automation of WGS may also reduce human error, as described above. Further, the same WGS can also provide information about potential transmission (34), and the same methods as used to identify resistance determinants could be used to bioinformatically extract the presence/absence of virulence genes (35).

The advances provided by WGS, combined with robust clinical outcome data, should greatly enhance our understanding of the genetic basis of antimicrobial resistance, with the potential for identifying new antimicrobial drug targets. The consequent promise of improved drug discovery in the face of current global concern regarding emerging antimicrobial resistance makes the prospect of routine use of WGS increasingly attractive.

## Supplementary Material

Supplemental material
