# Supplementary material for: Prediction of Staphylococcus aureus Antimicrobial Resistance by Whole-Genome Sequencing
Source: J Clin Microbiol. 2014 Apr;52(4):1182–91. doi: 10.1128/JCM.03117-13 (PMC3993491; doi:10.1128/JCM.03117-13)
Supplement: Supplemental material [file supp_52_4_1182__index.html]

Prediction of Staphylococcus aureus Antimicrobial Resistance by Whole-Genome Sequencing — Supplemental material 

# Prediction of Staphylococcus aureus Antimicrobial Resistance by Whole-Genome Sequencing

## Supplemental material

**Files in this Data Supplement:**

- Supplemental file 1 -

  Tables S1 (*fusA* variants associated with fusidic acid resistance), S2 (*dfrB* variants associated with resistance to trimethoprim), S3 (*rpoB* variants associated with resistance to rifampin), S4 (*grlA*, *gyrA*, and *grlB* variants associated with resistance to quinolones), and S5 (Frequency of variants across all study isolates)

  PDF, 587K
