# Supplementary material for: Prediction of Staphylococcus aureus Antimicrobial Resistance by Whole-Genome Sequencing
Source: J Clin Microbiol. 2014 Apr;52(4):1182–91. doi: 10.1128/JCM.03117-13 (PMC3993491; doi:10.1128/JCM.03117-13)
Supplement: Supplemental material [file JCM.03117-13_zjm999093277so1.pdf]

Supplementary data

Table S1. *fusA* variants associated with fusidic acid resistance

Amino acid substitutions	Reported MIC (mg/L)	References
B434N	>128	1
L461K	>128	2, 3, 4, 5
L461K+H457Q	>128	4
L461K+C473S	>128	4
H457Y+A67T	>128	3, 4
H457Y+A70V	>128	4
H457Y+R76C	>128	4
L461K+H457Q+A655V+V90I	>128	2
L461F+A376V +A655P+ D463G	>128	6
H457Q+L461F	128	4
V90I+H457Q+L461K	128	5
H457Y	48-96	1, 2, 3, 4, 5, 7, 8
L461S+P404L+A71V	96	3
L461S+V90I	16-64	7
H457Y +M161I	16-64	4
H457Y+S416F	64	2
H557Y +D373N	64	7
Q115L	48	1
T436I	8-48	1, 2
G452S	8-32	1, 2, 3
L456F+A376V	32	4
L461F+E444V	32	4
E444K	16	4
G556S	16	8
R659L	16	4
P404L	4-16	1, 2, 4, 7
G451V	3-16	3, 4
H457Y+R659L	16	8
H457Y+G556S	2-16	8
L461S+E233Q+V90I	16	7
A655E	12	1
G452C	12	1
P114H	12	1
R659C	12	1
R659S	12	1
H438N	8	1
P478S	8	4
R659H	8	1
A67T+P406L	8	2
A71V +P404L	8	4
F652S+Y654N	8	2
A71V + D189G+P406L	8	6
L456F	4-8	1, 2
M453I	4-8	4, 6

L461S	2-8	4, 5, 6, 7
R464C	2-8	1, 2
V90I	1-8	5, 6, 7
P406L	6	1
R464S	6	1
R464H	6	1
G617D	4	1
G664S	4	1
H457Q	4	7, 9
T385N	4	1
T656K	4	7
M651I	2	4
P404Q	2	7
Mutations where effect on MIC is not described / is less than EUCAST breakpoint		
A376V	Not described	5
F441Y	Not described	5
A70V+A160V+H457Y	Not described	5
V607I	0.19	3
V90A	Not described	5
T387I+E449K	Not described	5
D189V+L430S	Not described	5

Table S2: *dfrB* variants associated with resistance to trimethoprim

Amino acid substitutions	Reported MIC (mg/L)	Reference
F99Y+H31N	64	10
F99Y+H150R	64	10
F99Y+L21V+N60I	64	10
H31N	20	11
F99Y	16	10, 11, 12
H150R	8	12
F99S	4	12
L41F	4	12
F99I	2.5	11

Table S3: *rpoB* variants associated with resistance to rifampicin

Amino acid substitution	Reported MIC (mg/L)	References
Q468K	>1024	13, 14
D471Y+S486L	>1024	13
S486L	1024	13, 15
H481N+A473T+A477T	256-1024	13, 14, 16

H481N+I527M	>512	13, 14, 17
Q468L	512	13
H481N+Q565R+S529L	512	13, 14
H481N+L466S	256-512	13, 14
H481Y	128-512	13, 15, 16, 17, 18
H481N+S529L	128-512	13, 14, 16
H481N+I527M	>256	17
H481D+Q468K	>256	19
H481D+I527L	>256	19
H481D+S529L	>256	19
Q468R	256	18
Q456K	128-256	16
R484H	128-256	16, 18
H481D	2-256	16, 19
A477D	128	18
D550G	128	18
H481D+A477T	128	19
ins 475H	>32	15
ins475G	16	16
M470T+D471G	12	15
S463P	8	16
D471Y	2-6	13, 15, 18
S464P	4	18
I527F	4	16
H481N	1-4	13, 14, 16
A477V	1	18

Table S4: *grlA*, *gyrA* and *grlB* variants associated with resistance to quinolones

grlA amino acid substitution	*gyrA* amino acid substitution	*grlB* amino acid substitution	Reported MICs (mg/L)	References
S80F+E84V	E88K		>1024	20
S80Y+E84G	S84L+S85P		512-1024	21
	S84L		>512	22, 23, 24
	S84A		>512	23
S80F+E84L	S84L+S85P		512*	25
S80Y+E84G	S84L+E88K		256-512	21
S80Y+E84K	S84L+S85P		256-512*	25
	S84L+S85P		>256	22, 24
S80Y+E84G	S84L		256	21
S80F+S108N	S84L+S85P		256*	25
S80Y+E84G	S84L+E88L		256*	25
S80Y+E84K	S84L		128-256	26, 27
S80F+E84K	S84L		32-256	27, 28, 29

S80F+A48T	S84L		32-256	20, 30
S80F+E84K	S84L+S85P		32-256	27, 29
	S84L+E88K		>128	24
S80F+E84K			>128	24
S80F+E84G	S84L		128	28
S80Y	S84L		32-128*	26, 27
S80F	S84L		8-128*	21, 25, 27, 28, 29, 30, 31, 32
S80Y	E88K		8-128*	26
E84K	S84L		8-128*	25, 27, 28, 33
S80F			0.5-128	20, 24, 27
S80F	S84L+E88K		>100	33
S80F	S84L	P451S	>64*	26, 29
S80F+E84V	S84L		>64	29
S80F+Y83N	S84L		>64	29
S80F+E84K	S84L+E88G		>64	29
	S85P		64	22
S80F	S84L+S85P		64	29
S80F	S84L+S85P	D432V	64	29
S80F	S84L+S85P	D432N or H	64	21
A116E	S84L		32	32
S80F	S84L	P585S	32	29
	E88K		16-32	27
S80F	E88G		16-32	27
S80F	E88K		8-32*	20, 26, 27, 28, 33
S80F+D432G	S84L		16	20
D79V+S80Y	S84L		16*	26
S80F+V41G+I45M	S84L		16	20
S80F+V41G	S84L	E422D	16	20
S80F	S84L+G106D		16	20
S80Y	E88G		8-12.5	27, 33
E84K	S84L	D443E+R444S	8*	25
D79V+S80F	E88K		8*	26
	S84L	R470D	4	32
S80Y			0.5-4	24
S80F+I45M		E422D	2	20

*MICs are for levofloxacin

Table S5: Frequency of variants across all study isolates

Gene	Variant	Frequency
grlA	S80F	241
	S80Y	3
	E84G	2
	E84K	2
	I45M	52
grlB	E422D	421

	P451S	26
	D432V	4
	P585S	1
gyrA	S84L	238
	S84A	1
rpoB	H481Y	6
	A477D	1
	S464P	1
dfrB	F99Y	69
	H150R	6
	L21V	1
	L41F	1
	H31N	1
fusA	A67T	4
	L461K	8
	L461S	3
	P406L	4
	V90I	9
	H457Y	4
	H457Q	1
	G452S	2
	L461S	2
	L461F	1
	P404Q	1
	T656S	1

References

1. Norström T, Lannergård J, Hughes D. Genetic and phenotypic identification of fusidic acid-resistant mutants with the small-colony-variant phenotype in *Staphylococcus aureus* . *Antimicrob Agents Chemother* 2007; **51**:4438-46

2. Besier S, Ludwig A, Brade V, Wichelhaus TA. Molecular analysis of fusidic acid resistance in *Staphylococcus aureus* . *Mol Microbiol* 2003; **47**:463-9

3. O'Neill AJ, McLaws F, Kahlmeter G *et al*. Genetic basis of resistance to fusidic acid in staphylococci. *Antimicrob Agents Chemother* 2007; **51**:1737-40

4. Chen HJ, Hung WC, Tseng SP *et al.* Fusidic acid resistance determinants in *Staphylococcus aureus* clinical isolates. *Antimicrob Agents Chemother* 2010; **54**:4985-91

5. Castanheira M, Watters AA, Bell JM *et al.* Fusidic acid resistance rates and prevalence of resistance mechanisms among *Staphylococcus spp*. isolated in North America and Australia, 2007-2008. *Antimicrob Agents Chemother* 2010; **54**:3614-7

6. Lannergård J, Norström T, Hughes D. Genetic determinants of resistance to fusidic acid among clinical bacteremia isolates of *Staphylococcus aureus* . *Antimicrob Agents Chemother* 2009; **53**:2059-65

7. Chen CM, Huang M, Chen HF, Ke SC, Li CR, Wang JH, Wu LT. Fusidic acid resistance among clinical isolates of methicillin-resistant *Staphylococcus aureus* in a Taiwanese hospital. *BMC Microbiol* 2011; **11**:98

8. McLaws FB, Larsen AR, Skov RL *et al.* Distribution of fusidic acid resistance determinants in methicillin-resistant *Staphylococcus aureus* . *Antimicrob Agents Chemother* 2011; **55**:1173-6

9. Castanheira M, Watters AA, Mendes RE *et al.* Occurrence and molecular characterization of fusidic acid resistance mechanisms among *Staphylococcus spp.* from European countries (2008*). J Antimicrob Chemother* 2010; **65**:1353-8

10. Dale GE, Broger C, D'Arcy A *et al.* A single amino acid substitution in *Staphylococcus aureus* dihydrofolate reductase determines trimethoprim resistance*. J Mol Biol* 1997; **266**:23-30

11. Frey KM, Viswanathan K, Wright DL, Anderson AC. Prospective screening of novel antibacterial inhibitors of dihydrofolate reductase for mutational resistance. *Antimicrob Agents Chemother* 2012; **56**:3556-62

12. Vickers AA, Potter NJ, Fishwick CW *et al.* Analysis of mutational resistance to trimethoprim in *Staphylococcus aureus* by genetic and structural modelling techniques*. J Antimicrob Chemother* 2009; **63**:1112-1117

13. Wichelhaus T, Schäfer V, Brade V. Differential effect of *rpoB* mutations on antibacterial activities of rifampicin and KRM-1648 against *Staphylococcus aureus* . *J Antimicrob Chemother* 2001; **47**:153-6

14. Wichelhaus T, Schäfer V, Brade V. Molecular characterization of *rpoB* mutations conferring cross-resistance to rifamycins on Methicillin-resistant *Staphylococcus aureus* . *Antimicrob Agents Chemother* 1999; **43**:2813-6

15. Hellmark B, Söderquist B, Unemo M. Simultaneous species identification and detection of rifampicin resistance in staphylococci by sequencing of the *rpoB* gene. *Eur J Clin Microbiol Infect Dis* 2009; **28**:183-90

16. Villar M, Marimón JM, García-Arenzana JM *et al*. Epidemiological and molecular aspects of rifampicin-resistant *Staphylococcus aureus* isolated from wounds, blood and respiratory samples*. J Antimicrob Chemother* 2011; **66**:997-1000

17. Van Rensburg MJ, Whitelaw AC, Elisha BG. Genetic basis of rifampicin resistance in Methicillin-resistant *Staphylococcus aureus* suggests clonal expansion in hospitals in Cape Town, South Africa. *BMC Microbiol* 2012; **12**:46

18. Aubry-Damon H, Soussy CJ, Courvilin P. Characterization of mutations in the *rpoB* gene that confer rifampin resistance in *Staphylococcus aureus* . *Antimicrob Agents Chemother* 1998; **42**:2590-4

19. Mick V, Domínguez MA, Tubau F. Molecular characterization of resistance to Rifampicin in an emerging hospital-associated Methicillin-resistant *Staphylococcus aureus* clone ST228, Spain. *BMC Microbiol* 2010; **10**:68

20. Schmitz FJ, Jones ME, Hofmann B *et al*. Characterization of *grlA*, *grlB*, *gyrA*, and gyrB mutations in 116 unrelated isolates of *Staphylococcus aureus* and effects of mutations on ciprofloxacin MIC. *Antimicrob Agents Chemother* 1998; **42**:1249-52

21. Sanfilippo CM, Hesje CK, Haas W, Morris TW. Topoisomerase mutations that are associated with high-level resistance to earlier fluoroquinolones in *Staphylococcus aureus* have less effect on the antibacterial activity of besifloxacin. *Chemotherapy* 2011; **57**):363-71

22. Sreedharan S, Oram M, Jensen B *et al.* DNA gyrAse *gyrA* mutations in ciprofloxacin-resistant strains of *Staphylococcus aureus* : close similarity with quinolone resistance mutations in *Escherichia coli*. *J Bacteriol* 1990; **172**:7260-2

23. Peterson LR, Willard KE, Sinn *LM et al. GyrA* sequence analysis of *Staphylococcus aureus* and methicillin-resistant *S. aureus* strains selected, in vitro, for high-level ciprofloxacin resistance. *Diagn Microbiol Infect Dis* 1993; **17**:97-101

24. Takahashi H, Kikuchi T, Shoji S *et al.* Characterization of *gyrA*, gyrB, *grlA* and *grlB* mutations in fluoroquinolone-resistant clinical isolates of *Staphylococcus aureus* . *J Antimicrob Chemother* 1998; **41**:49-57

25. Emerging multiple mutations and high-level fluoroquinolone resistance in methicillin-resistant *Staphylococcus aureus* isolated from ocular infections. Iihara H, Suzuki T, Kawamura Y, Ohkusu K, Inoue Y, Zhang W, Monir Shah M, Katagiri Y, Ohashi Y, Ezaki T. *Diagn Microbiol Infect Dis* 2006; **56**:297-303

26. Horii T, Suzuki Y, Monji A *et al. D*etection of mutations in quinolone resistance-determining regions in levofloxacin- and methicillin-resistant *Staphylococcus aureus* : effects of the mutations on fluoroquinolone MICs. *Diagn Microbiol Infect Dis* 2003; 46:139-45

27. Noguchi N, Okihara T, Namiki Y *et al.* Susceptibility and resistance genes to fluoroquinolones in methicillin-resistant *Staphylococcus aureus* isolated in 2002. *Int J Antimicrob Agents* 2005; 25:374-9

28. Costa SS, Falcão C, Viveiros M *et al.* Exploring the contribution of efflux on the resistance to fluoroquinolones in clinical isolates of *Staphylococcus aureus* . *BMC Microbiol* 2011; **11**:241

29. Yoon EJ, Lee CY, Shim MJ *et al.* Extended spectrum of quinolone resistance, even to a potential latter third-generation agent, as a result of a minimum of two *GrlA* and two *GyrA* alterations in quinolone-resistant *Staphylococcus aureus. Chemotherapy* 2010; **56**:153-7

30. Schmitz FJ, Fluit AC, Hafner D *et al.* Development of resistance to ciprofloxacin, rifampicin, and mupirocin in methicillin-susceptible and -resistant *Staphylococcus aureus* isolates. *Antimicrob Agents Chemother* 2000; **44**:3229-31

31. Schmitz FJ, Hofmann B, Hansen B *et al.* Relationship between ciprofloxacin, ofloxacin, levofloxacin, sparfloxacin and moxifloxacin (BAY 12-8039) MICs and mutations in *grlA*, *grlB*, *gyrA* and

gyrB in 116 unrelated clinical isolates of *Staphylococcus aureus* . *J Antimicrob Chemother* 1998; **41**:481-4

32. Ince D, Hooper DC. Mechanisms and frequency of resistance to premafloxacin in *Staphylococcus aureus* : novel mutations suggest novel drug-target interactions. *Antimicrob Agents Chemother* 2000; 44:3344-50

33. Takahata M, Yonezawa M, Kurose *et al.* Mutations in the *gyrA* and *grlA* genes of quinolone-resistant clinical isolates of methicillin-resistant *Staphylococcus aureus* . *J Antimicrob Chemother* 1996; **38**:543-6
